# An Algorithm for Precipitation Correction in Flood Season Based on Dendritic Neural Network

**DOI:** 10.3389/fpls.2022.862558

**Published:** 2022-05-24

**Authors:** Tao Li, Chenwei Qiao, Lina Wang, Jie Chen, Yongjun Ren

**Affiliations:** ^1^School of Artificial Intelligence, Nanjing University of Information Science and Technology, Nanjing, China; ^2^School of Electronics and Information Engineering, Nanjing University of Information Science and Technology, Nanjing, China; ^3^School of Computer Software, Nanjing University of Information Science and Technology, Nanjing, China

**Keywords:** machine learning, summer precipitation correction, dendrite net (DD) and artificial neural networks (ANN), mean square error (MSE), temporal correlation coefficient (TCC), anomaly correlation coefficient (ACC)

## Abstract

In recent years, the National Climate Center has developed a dynamic downscaling prediction technology based on the Climate-Weather Research and Forecasting (CWRF) regional climate model and used it for summer precipitation prediction, but there are certain deviations, and it is difficult to predict more accurately. The CWRF model simulates the summer precipitation forecast data from 1996 to 2019 and uses a combination of dendrite net (DD) and artificial neural networks (ANNs) to conduct a comparative analysis of summer precipitation correction techniques. While summarizing the characteristics and current situation of summer precipitation in the whole country, the meteorological elements related to precipitation are analyzed. CWRF is used to simulate summer precipitation and actual observation precipitation data to establish a model to correct the precipitation. By comparing with the measured data of the ground station after quality control, the relevant evaluation index analysis is used to determine the best revised model. The results show that the correction effect based on the dendritic neural network algorithm is better than the CWRF historical return, in which, the anomaly correlation coefficient (ACC) and the temporal correlation coefficient (TCC) both increased by 0.1, the mean square error (MSE) dropped by about 26%, and the overall trend anomaly (Ps) test score was also improved, showing that the machine learning algorithms can correct the summer precipitation in the CWRF regional climate model to a certain extent and improve the accuracy of weather forecasts.

## Introduction

Climate prediction is the process of predicting the likely trend of climate development in the future based on the changing laws of the past climate. In recent years, the forward-looking role of climate prediction in disaster prevention and mitigation has been recognized more and more, and the demand for prediction in all walks of life is increasing. With the needs of social and economic development, research on climate prediction needs to be improved urgently (Yingdong et al., 2011; Bannister et al., 2019; Xinmin et al., 2019). Accurate precipitation data are essential for understanding climate change and associated hydrological responses from small basins to large regions around the world (Pan et al., 2016). At present, global and regional climate models are the primary tools for climate change simulation and prediction research (Rummukainen, 2010), but they are restricted by the complexity of the climate system and the level of scientific development. Compared with actual observations, climate model simulations always show deviations in precipitation (Ren and Li, 2007; Kim et al., 2015, 2020).

Precipitation correction is an effective way to improve model forecasts. The concept of precipitation correction is proposed because there is some model data for flood season precipitation forecast in the current climate forecast business, but there are certain deviations. Therefore, it is hoped that the correction will reduce the error and improve the precipitation forecast accuracy and performance (Yao et al., 2017). With the development of weather forecasting technology, artificial intelligence, and data mining research, the use of intelligent computing and data mining technology to correct regional precipitation provides a new and effective method for improving the existing precipitation forecast quality and prediction accuracy, which has become one of the research hotspots.

The dendritic neural network has achieved great success in many fields (Wu et al., 2009, 2018; Kisi et al., 2017; Egrioglu et al., 2019; Xue et al., 2021; Achite et al., 2022). The diverse kinds of synaptic plasticity and non-linearity mechanisms enable synapses to take a valuable part in calculation (Gao et al., 2018). Synaptic non-linearity is implemented in a dendritic structure to effectively solve linearly inseparable problems, and this model has been applied to a variety of complex continuous functions (Zhou et al., 2016; Chen et al., 2017; Ji et al., 2019, 2021). Among the various types of soft computing approaches, the artificial neural networks (ANNs) models have satisfactorily been applied to non-linear hydrologic simulations such as rainfall (Acharya et al., 2014), evapotranspiration (Kisi et al., 2015), and river flow (Zounemat-Kermani et al., 2013). In recent years, related research has also been carried out at home and abroad. For example, Huating Xu (Xu et al., 2018) mentioned that the global environmental multiscale (GEM) model is widely used as a high-resolution medium-term prediction model for precipitation forecasting in various parts of Canada. With the continuous deepening of regional climate simulation research, the new generation of regional climate models, Climate-Weather Research and Forecasting (CWRF), has begun to be widely used because of its excellent performance (Guanzhou and XinZhong, 2017). For example, Xiaoyun et al. (2019) used the CWRF regional model to propose a cumulative probability transformation deviation correction method for extreme precipitation to test and evaluate its applicability to extreme precipitation correction. For example, Zhang and Zhi (2018) proposed using the frequency matching method (FMM) to calibrate the large-scale precipitation forecast data obtained from the Public Meteorological Service Center of the China Meteorological Administration (CMA). The results show that FMM calibration can significantly improve the forecasting skills of large-scale precipitation forecasts. For example, Jixue et al. (2016) used 18 meteorological elements, such as temperature, humidity, pressure, and wind field, in high-altitude weather observation data to train a three-layer BP neural network model. The experimental results show that the ANN has good application prospects in short-term precipitation forecasting. For example, Yuting et al. (2020) trained the ANN model with the annual precipitation of five stations in the western area of Taihu Lake. The results showed that the fitting and prediction accuracy and stability of the ANN model based on component analysis were higher than those of the original ANN and linear autoregression models and the other 4 types of neural networks. For example, Liu and Wang (2021) proposed a white-box dendrite net (DD) with a logical operational relationship, while the ANN network is a black-box network that does not consider the fuzzy non-linear mapping of the logical operation. DD has better generalization. Li et al. (2020) used the integrated network model of ANN and DD, and through digital recognition tasks, experiments proved the potential of artificial DDs to improve overall performance. Liangmin et al. (2016) proposed an objective clustering method based on nearest neighbor propagation to divide the climate of summer rainfall in China while using factors such as sea temperature and sea level pressure to establish a least-squares regression method to predict precipitation. Moreover, Chow and Cho (1997) described the development of new approaches to rainfall forecasting using ANN (Egrioglu et al., 2019).

In this article, the artificial DD is used to correct the CWRF simulation of summer precipitation so as to improve the accuracy of the CWRF prediction of precipitation. The data mining correlation algorithm is used for the correction of precipitation forecast results, and a precipitation correction scheme based on NN is proposed, which is used for the correction of precipitation in the summer flood season in China.

## Data and Methods

### Data Sources

The data used were obtained from the historical return results of the CWRF regional climate model (Climate Extension of WRF) (30 km resolution) in the summer of China (June–August) from 1991 to 2020 in the National Climate Center. Among them, 7 physical configuration combinations (i.e., case 01, 02, 06, 15, 16, 23, and 28) are selected from the four starting times (i.e., 00, 06, 12, and 18) on March 2 each year, a total of 28 samples; at the same time, the actual observation data (OBS) data from the ground station during the summer of 1991–2019 is selected as the target. The main meteorological elements included are precipitation, wind speed (10 m), wind volume (10 m), relative humidity (2 m), temperature (2 m), 500 altitude field, sea level pressure, whole layer water vapor, and vertical speed.

### Method Introduction

#### Artificial Neural Network Algorithm Model

Artificial neural network is a system with non-linear and adaptive information processing capabilities composed of a large number of neurons connected by different weights. ANN is a model that simulates the biological nervous system, which can approximate any non-linear function. There are three kinds of neurons, namely, (a) output neurons, those that send data out of the network; (b) input neurons, which receive external data; and (c) hidden neurons, whose signals remain in the ANN and join the input layer neurons to the neurons of the output layer (Samani et al., 2007; Zounemat-Kermani, 2012). Traditional precipitation forecasting and correction methods need to be analyzed by understanding precipitation principles and related influencing factors, while the ANN to achieve interannual precipitation forecasting does not need to be clear about the precipitation mechanism, and a model can be learned by learning precipitation and related element data to forecast future precipitation.

##### Artificial Neural Network Model


$$W\,h\,e\,r\,e\,\text{X}=\left[{\text{x}}_{1},{\text{x}}_{2},\ldots ,{\text{x}}_{n}\right],\text{W}=\left[\begin{array}{c} {\text{w}}_{\mathrm{i1}}\\ {\text{w}}_{\mathrm{i2}}\\ \cdot \\ {\text{w}}_{\mathrm{in}} \end{array}\right],\text{b}=\left[\begin{array}{c} {\text{b}}_{\mathrm{i1}}\\ {\text{b}}_{\mathrm{i2}}\\ \cdot \\ {\text{b}}_{i\,n} \end{array}\right]$$

**FIGURE 1:**
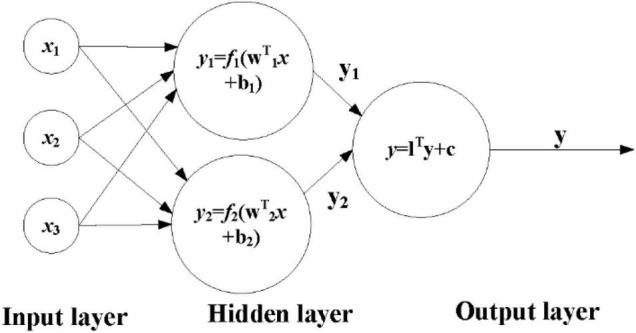
Flowchart of artificial neural network model.

The ANN algorithm model includes two parts, namely, forward propagation of information and back propagation of error. In Figure 1, **X_1_**∼**X_2_** are the input characteristic signals passed in from the neuron. **W_i1_**∼**W**_**i***n*_ are the weights corresponding to the incoming signals of different neurons. **b_i1_**∼**b**_**i***n*_ represent a bias. The setting of bias is to achieve accurate output and is an important parameter in the model. Different neurons are combined into the final input signal through different weight matrices. In Figure 1, **f**(*), **f** are called activation functions. The activation function mainly acts on the linear connection, and the non-linear function is added to the model, which can well realize the learning of non-linear problems. In the figure, **y** is the final output of the neuron.

##### Error Back-Propagation and Improvement

Back-propagation (BP) neural network is a process of continuous repetition when training the network, by collecting the errors generated by the system, returning these errors to the output value, and then using these errors to adjust the weight of the neurons so that the loss of the model propagates along the direction of the negative gradient. The parameters that affect the performance of the BP neural network mainly include the number of hidden layer nodes, the choice of activation function, and the choice of the learning rate. This article is based on the number of hidden layer nodes of the neural network to improve. According to the empirical formula:


(1) $$\begin{array}{c} \sqrt{\text{N}}+\text{X} \end{array}$$

Among them, **N** represents the number of sample features, and the value range of **X** is 1–10. The number of hidden layer nodes is determined step by step, and the prediction performance of each model is obtained by comparing different numbers of nodes, and the number of nodes with the best effect is selected as the number of hidden layer neurons. When determining the number of hidden layer nodes, the following conditions must be met: First, the number of hidden layer nodes must be less than **N**−**1**, that is, less than the number of input features. Otherwise, the system error of the network model is independent of the characteristics of the training sample and tends to be zero, i.e., the constructed network model has no generalization ability. Second, the number of training samples must be more than the connection weight of the network model, generally 2–10 times. Otherwise, the sample must be divided into several parts and the method of “training in turn” can be used to obtain a reliable neural network model.

#### Artificial Dendrite Net Algorithm Model

##### Dendrite Net Algorithm Model

The main concept of the DD model is that if the output logical expression contains the logical relationship of the corresponding class between the inputs (and\or\not), the algorithm can identify the class after learning. The white box machine learning algorithm DD shows excellent system recognition performance for the black box system. The DD has white box properties, controllable accuracy, better generalization ability, and lower computational complexity. Not only can DD be used in general engineering but as a module of deep learning, it also has broad development potential. The expression of the DD module is as follows:


(2) $${\text{A}}^{l}={\text{W}}^{l,l-1}\,{\text{A}}^{l-1}{}^{\circ}\text{X}$$

Among them, **A^l^**−**^1^** and **A^l^** represent the input and output of the model, **X** represents the input of DD, **W^l^**,^**l**−**1**^ is the weight matrix from module **l**−**1** to module **l**, and ° is the multiplication of corresponding elements, sometimes called Hadamard product.

##### Artificial Dendrite Net Algorithm Model

**FIGURE 2:**
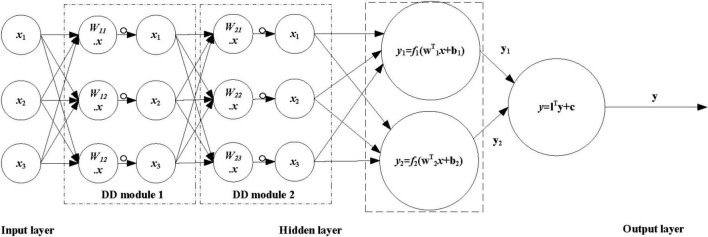
Flowchart of artificial dendritic neural network model.

The artificial dendritic neural network model is shown in Figure 2. The ANN algorithm model adds a DD module on the basis of ANN. The connection of different DD modules enhances the processing ability of neurons carrying information. The number of modules can effectively adjust the logic expression ability of DD and avoid excessive simulation. Together, it is easy to obtain models with outstanding generalization capabilities. By analyzing the existing ANN network model, it does not consider that the logical operation is a fuzzy non-linear mapping relationship, and the DD considers that the logical operation can converge to the global optimum with a high probability, so the integration of DD and ANN is adopted. The network model of the ANN adds a dendritic module to the hidden layer of the ANN to improve the generalization ability of the ANN algorithm model.

#### Evaluation Index

In this article, three indicators such as mean square error (MSE), temporal correlation coefficient (TCC), and spatial anomaly correlation coefficient (ACC), commonly used in meteorological services are used to evaluate the effect of machine learning on the correction of CWRF summer precipitation. The definitions are, respectively, as follows:

(1) The MSE is often used as an indicator to evaluate the prediction results of a machine learning model. The formula is as follows:

(3) $$MSE=\frac{1}{\text{N}}\sum_{i=1}^{N}{\left({\text{pre}}_{i}-{\text{obs}}_{i}\right)}^{2}$$

(2) The time correlation coefficient (TCC) can better represent the model’s ability to predict the abnormality of each grid point in a statistical sense and obtain a complete spatial distribution of correlation techniques. When calculating TCC, the mean square deviation and covariance of each grid point are required. The formula is as follows:

(4) $$\text{TCC}=\frac{\sum_{i=1}^{N}\left({\text{pre}}_{i}-\bar{{\text{pre}}_{i}}\right)\,\left({\text{obs}}_{i}-\bar{{\text{obs}}_{i}}\right)}{\sqrt{\sum_{i=1}^{N}{\left({\text{pre}}_{i}-\bar{{\text{pre}}_{i}}\right)}^{2}\,\sum_{i=1}^{N}{\left({\text{obs}}_{i}-\bar{{\text{obs}}_{i}}\right)}^{2}}}$$

In the formula, **pre_i_** and $\bar{{\text{pre}}_{i}}$ are the model return value or precipitation data model forecast value of **i** sample point and its multiyear average value; **obs_i_** and $\bar{{\text{obs}}_{i}}$ are the actual observation values of the precipitation data of sample point **i**; **N** is the total number of grid points actually participating in the evaluation.
(3) The ACC mainly reflects the degree of similarity between the forecasted value and the actual value of the space type and can also be called the spatial similarity coefficient. The spatial similarity coefficient can be calculated for each forecast field. The formula for calculating ACC is as follows:

(5) $$\text{ACC}=\frac{\sum_{i=1}^{N}\left(\Delta \,{\text{R}}_{f}-\bar{\Delta \,{\text{R}}_{f}}\right)\,\left(\Delta \,{\text{R}}_{o}-\bar{\Delta \,{\text{R}}_{o}}\right)}{\sqrt{\sum_{i=1}^{N}{\left(\Delta \,{\text{R}}_{f}-\bar{\Delta \,{\text{R}}_{f}}\right)}^{2}\,\sum_{i=1}^{N}{\left(\Delta \,{\text{R}}_{o}-\bar{\Delta \,{\text{R}}_{o}}\right)}^{2}}}$$

In the formula, **ΔR_f_** and $\bar{\Delta \,{\text{R}}_{f}}$ are the forecast value and multiyear average value of precipitation anomaly percentage ((actual measured value-historical average value of the same period)/historical average value of the same period); **ΔR_o_** and $\bar{\Delta \,{\text{R}}_{o}}$ are the corresponding observation values; **N** is the total number of stations actually participating in the evaluation.

The formula for the abnormal comprehensive (Ps) test score, which is a commonly used predictive scoring index in business, is:


(6) $${\text{P}}_{S}=\frac{\text{a}*\text{N0}+\text{b}*\text{N1}+\text{c}*\text{N2}}{\left(\text{N}-\text{N0}\right)+\text{a}*\text{N0}+\text{b}*\text{N1}+\text{c}*\text{N2}+\text{M}}*100$$

The scoring steps are as follows:

1. Determine whether the forecasted trend is correct from station to station and calculate the total number of stations with correct trend prediction **N0**;
2. Determine whether the first-level anomaly prediction is correct from station to station and calculate the total number of stations **N1** with the correct first-level anomaly prediction;
3. Determine whether the second-level anomaly prediction is correct from station by station and count the total number of stations **N2** with the correct second-level anomaly prediction;
4. The number of stations where the percentage of precipitation anomaly is ≥ 100% or equal to −100%, and the temperature anomaly is ≥ 3°C or ≤ −3°C (referred to as missed stations, denoted as **M**) without a second-level anomaly forecast;
5. Count the number of stations **N** that actually participated in the assessment, that is, the number of stations that are required to participate in the assessment minus the number of stations that are not in the live test;

**a**, **b**, and **c** are the weight coefficients of the climate trend item, the first-level anomaly item, and the second-level anomaly item, respectively. This algorithm takes **a**=**2**, **b**=**2**, and **c**=**2**, respectively.

## Precipitation Correction Model Construction

### National Climate Division

In this study, the precipitation prediction results of the CWRF model in the flood season (June–August) in China (8.37°N–58.75°N, 58.40°E–161.60°E) were selected as the target of forecast correction. The geographical diversity of climate is obvious, which makes it difficult for a single model to represent the climate characteristics of the entire China, leading to certain difficulties in climate forecasting. To realize the correction of the national regional precipitation forecast data, regional modeling forecasts are carried out for the whole country. That is, according to the climatic characteristics of different regions, they establish model algorithms suitable for their respective climatic characteristics, promote the high generalization ability of the model, and improve the accuracy of precipitation forecasting. According to the climate characteristics of different regions, China is divided into eight regions (Wang and Yang, 2017). The specific results are shown in Table 1.

**TABLE 1:** Eight area names.

Abbreviations	Full name
NWCH	Northwest China
TP	Tibetan Plateau
BBYR	Big Bend of Yellow River
SWCH	Southwest China
NECH	Northeast China
NCH	North China
YHRB	Yangtze–Huaihe River Basin
SCH	South China

**FIGURE 3:**
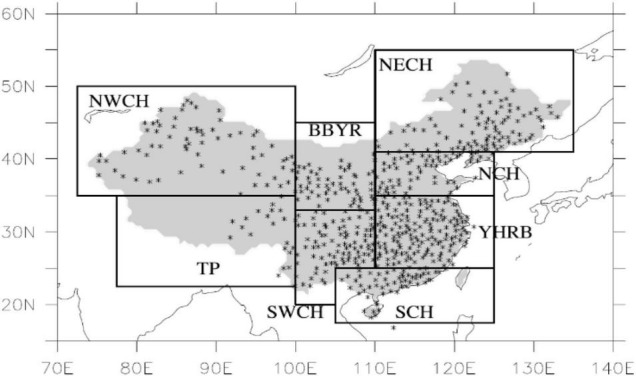
China’s terrestrial climate regional division map.

In the precipitation data file, the data size is 231 × 171, that is, 231 × 171 grid points, including the China region. Now, the regions are divided according to Figure 3 and Table 1.

### Feature Selection and Data Organization

#### Feature Selection

Pearson correlation coefficient (PCC) and random forest algorithm for feature selection comparison.

Pearson correlation coefficient, also known as Pearson product-moment correlation coefficient (PPMCC or PCCs), is used to measure the correlation between two variables **X** and **Y** (linear correlation), and its value is between -1 and 1.

The PCC between two variables is defined as the quotient of the covariance and standard deviation between the two variables:


(7) $$\rho_{X,Y}=\frac{\text{cov}\,\left(X,Y\right)}{\sigma_{X}\,\sigma_{Y}}=\frac{\text{E}\,\left[\left(\text{X}-\mu_{X}\right)\,\left(\text{Y}-\mu_{Y}\right)\right]}{\sigma_{X}\,\sigma_{Y}}$$

The above formula defines the overall correlation coefficient, and Greek lowercase letters are commonly used as representative symbols. Estimate the covariance and standard deviation of the sample to get the PCC. Commonly used English lowercase letter *r* stands for:


(8) $$\text{r}=\frac{\sum_{i=1}^{n}\left({\text{X}}_{i}-\bar{\text{X}}\right)\,\left({\text{Y}}_{i}-\bar{\text{Y}}\right)}{\sqrt{\sum_{i=1}^{n}{\left({\text{X}}_{i}-\bar{\text{X}}\right)}^{2}}\,\sqrt{\sum_{i=1}^{n}{\left({\text{Y}}_{i}-\bar{\text{Y}}\right)}^{2}}}$$

**r** can also be estimated from the mean value of the standard scores of the sample points (**X_i_**,**Y_i_**), and an expression equivalent to the above equation can be obtained:


(9) $$\text{r}=\frac{1}{\text{n}-1}\,\sum_{i=1}^{n}\left(\frac{{\text{X}}_{i}-\bar{\text{X}}}{\sigma_{X}}\right)\,\left(\frac{{\text{Y}}_{i}-\bar{\text{Y}}}{\sigma_{Y}}\right)$$

Among them, $\frac{X_{i}-\bar{X}}{\sigma_{X}}$, $\bar{\text{X}}$, and σ**_X_** are the standard score, sample mean, and sample standard deviation of **X_i_** sample, respectively.

1. Use PCC to select features for precipitation data.
2. Use random forest model to select features.

After experiments, for the random forest model, using the attribute column obtained through the PCC in step 1 for training, the score is 0.97; while using the features selected by the random forest for training, the score is 0.98. It can be seen that the use of random forest for feature selection still has a certain effect on improving the ability of the model on this dataset.

Later, we picked the characteristics that are most important to us: the precipitation and the historical return of the CWRF. These are cumulative wind volume v component, cumulative wind speed, cumulative temperature 2 m, and precipitation correlation of the historical return.

#### Data Organization

First of all, considering the influence of the age average on forecasting, anomalies are commonly used in meteorological forecasting operations, so this article also chooses anomalies to preprocess the data. Taking precipitation as an example, comparing the rainfall forecasted by the model with the average rainfall over the years, the forecast is the value of the forecast rainfall minus the average rainfall over the same period. It is generally used in medium- and long-term forecasts and can be used as a reference for flood control and drought resistance. The historical year-month (year) precipitation distance is equal to the difference between the historical year-month (year) precipitation and the cumulative year-month (year) average precipitation.


(10) Dpt=Pre-AvgPre

Among them, Pre represents the precipitation of a certain location in a certain month in a certain year, AvgPre represents the average precipitation of a certain location in the historical years that have been recorded at that point, and Dpt represents the precipitation of a certain location in a certain year from the average value in a certain month. If Dpt < **0**, it is a positive anomaly, and the annual precipitation at the location is greater than the cumulative annual average precipitation; if Dpt > **0**, it is a negative anomaly, and the annual precipitation at the location is less than the cumulative annual average precipitation.

Then, the summer precipitation data are gridded data. Each grid point has only the CWRF return data and actual observation data at a specific time and a specific location. Based on the similarity characteristics of the climate in the neighboring areas, the target point is divided into small areas of M*M, and then each grid point has M*M feature data; (1) the precipitation is corrected by the single-element integration method: the value of M is 3, which is a small area of 3 × 3 around the grid point. Second, taking into account the influence of the precipitation months in summer (6–8), the average precipitation anomaly of the past 5 months (4–8) is used as the input feature of the model to organize the data. That is, the April–August precipitation anomaly and the average precipitation of the 3 × 3 grid points around the current year of the CWRF model precipitation are used as the characteristics of the model training input, and the output is the precipitation anomaly from April to August of the current year; (2) using the multifactor integration method to correct the precipitation: select the historically reported precipitation of CWRF, the cumulative wind volume u component, the cumulative wind volume v component, the cumulative wind speed, and the cumulative temperature 2 m. The data are preprocessed and converted into monthly anomaly data. Five meteorological elements, input according to 3 × 3 grid points are selected; the input data from June to August (or a single month) are organized, and the corresponding 1-month precipitation anomaly is delivered. Data according to the above different methods are organized, and a data format suitable for network model training is constructed. Finally, based on the interdecadal influence in the meteorological field, this article uses the data of nearly **N** years when training based on the artificial DD model and then predicts the summer precipitation in **N1** years. The range of **N** selected in this article is 3–10. The analysis of the experimental comparison results shows that **N** is set to 5, that is, the training algorithm model using the data of the past 5 years is better than the correction results of other years.

**FIGURE 4:**
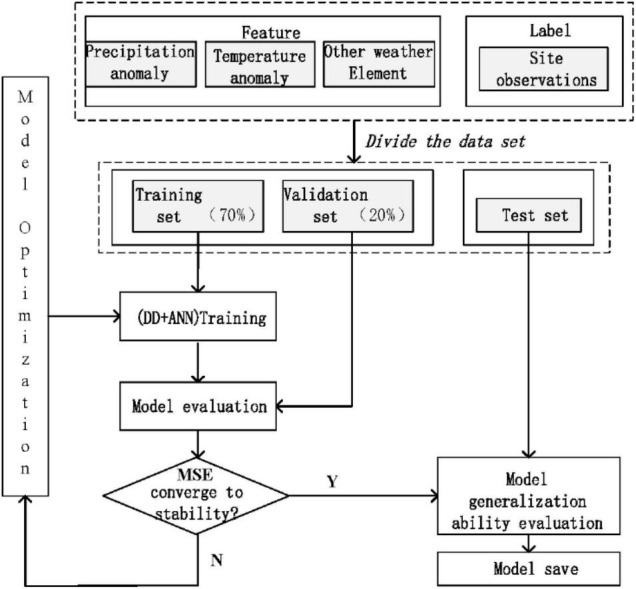
Flowchart of summer precipitation correction algorithm based on artificial dendrite net.

Through the above-mentioned modeling method, the forecast factors are optimized at the same time for the 28 sample forecast data of 7 different physical parameter configurations and 4 start times. One or more forecast factors are used to predict the precipitation, and the 28 sample forecast results use the weighted average method to integrate as the final output. First, the organized data are divided as follows: 70% of the data as the training set, 20% of the data as the validation set, and the rest as the test set. At the same time, the MSE is selected as the error loss function for network model training, based on the artificial DD model summer precipitation. The flowchart is shown in Figure 4.

## Analysis of Precipitation Correction Results

### Regional Precipitation Forecast Revision

(1) Comparison of TCC before and after the regional correction in China.

**FIGURE 5:**
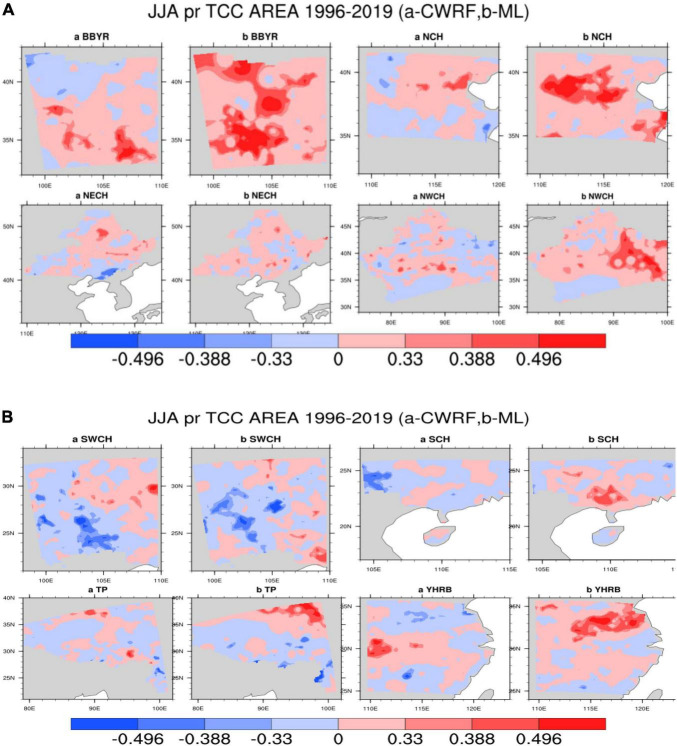
**(A)** Comparison of regional precipitation time correlation coefficients [Climate-Weather Research and Forecasting (CWRF) vs. Machine Learning (ML)], the color code numbers 0.33, 0.388, and 0.496, respectively, represent that the correlation coefficients have passed the significance test of 90%, 95%, and 99%, respectively. **(B)** Comparison of regional precipitation time correlation coefficients (CWRF vs. ML); the color code numbers are the same as panel **(A)**.

The eight sub-areas are divided to calculate the TCC of each grid point, and the correction results of the precipitation forecast of the flood season model are tested. Figures 5A,B shows the TCC comparison test of precipitation and actual observed precipitation (OBS) in the eight sub-regions during the flood season from 1996 to 2019. It can be seen from Figures 5A,B that after the machine learning method is revised, the positive value range of the TCC of precipitation prediction in different regions has been improved to varying degrees, especially in the eastern key areas of the rain belt during the flood season in China [South China (SCH), Yangtze-Huaihe River Basin (YHRB), North China (NCH), Northeast China (NECH)] TCC correction effect is more obvious. The YHRB, the NECH, and the Southwest Region (SWCH) have significantly improved TCC compared with other regions. After the revision, the regions that passed the 90% significance test significantly increased.

**FIGURE 6:**
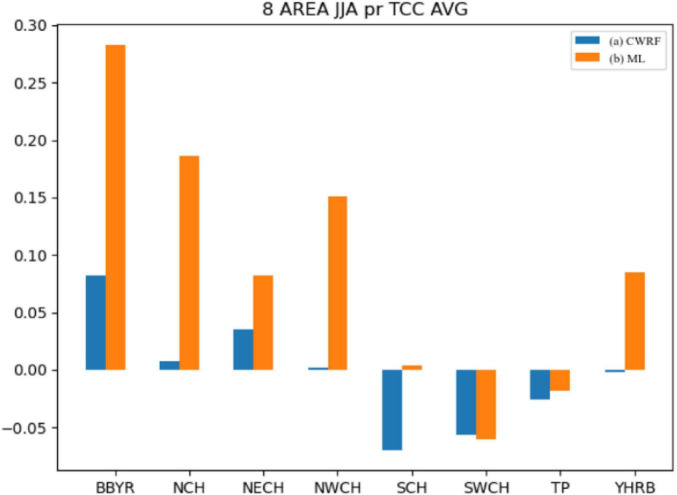
Comparison of mean values of regional precipitation time correlation coefficients (CWRF vs. ML). (a) CWRF prediction and (b) machine learning correction.

It can be seen from Figure 6 that after the correction of the machine learning method, the mean value of the TCC of the precipitation prediction in different regions has been improved to different degrees. The range of the positive value after the correction by the improved ANN algorithm has been improved more obviously, especially in the rainy season in China. The effect of TCC correction in key eastern regions of the belt [Big Bend of Yellow River (BBYR), Northwest China (NWCH), SCH, YHRB, NCH, and NECH] is more obvious. The Jianghuai River Basin (YHRB), BBYR, NCH, and NWCH have significantly improved TCC compared with other regions. From the regional average, the difference between the results of experiments before and after the correction has more than 0.2.

(2) Comparison of spatial correlation coefficients (i.e., ACC) before and after the revision of China’s regional precipitation forecasts.

**FIGURE 7:**
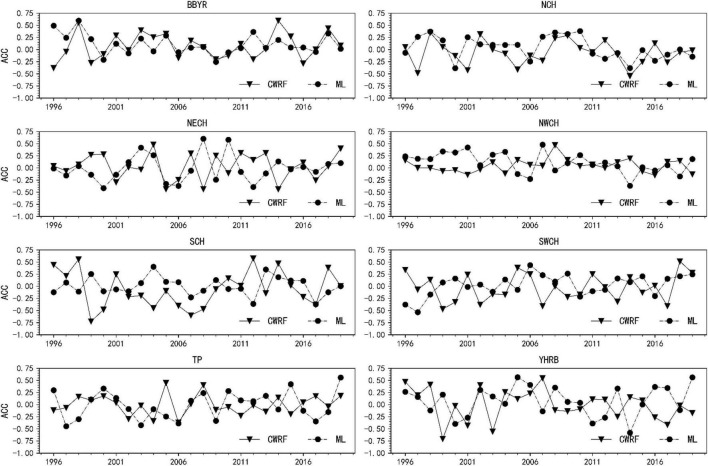
Comparison of anomaly correlation coefficient (ACC) before and after precipitation correction in 8 sub-regions.

Calculate the spatial correlation coefficients between the precipitation during the flood season from 1996 to 2019 and the actual observed precipitation for the eight sub-regions divided, and the ACC values before and after the correction are shown in Figure 7. It can be seen from Figure 7 that the spatial correlation coefficient of the corrected results of ML in the 8 regions is larger than the original prediction of CWRF. The spatial correlation coefficient of b is relatively close. After the machine learning method is revised, the spatial correlation coefficients of precipitation forecasts in different regions have been improved to varying degrees. The algorithm has certain predictive performance.

**FIGURE 8:**
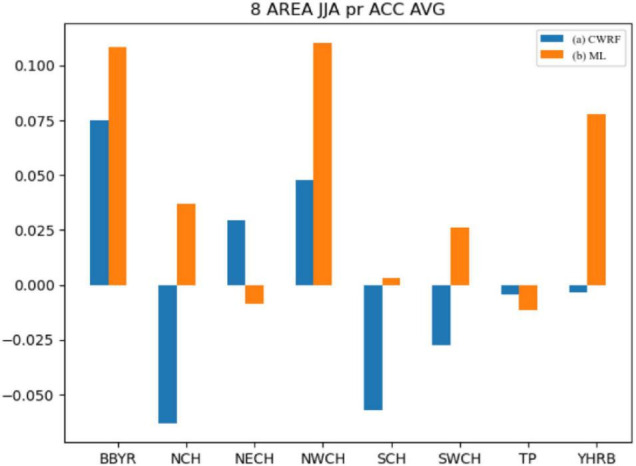
Comparison of mean values of spatial correlation coefficients of regional precipitation (CWRF vs. ML). (a) CWRF prediction and (b) machine learning correction.

It can be seen from Figure 8 that after the correction of the machine learning method, the mean values of the spatial correlation coefficients of precipitation forecasts in different regions have improved to varying degrees. Among them, BBYR, NCH, NWCH, SCH, SWCH, and YHRB have improved significantly, especially during the flood season in China. The ACC correction effect in the key eastern areas of the rain belt (i.e., SCH, YHRB, NCH, NECH, and SWCH) is more obvious. The Jianghuai River Basin (YHRB), the NECH, and the Southwest Region (SWCH) have significantly improved ACC compared with other regions. From the regional average, the difference in results before and after the correction has risen more than 0.1 on average.

### Evaluation of the Effect of the National Precipitation Forecast Revision

(1) Comparison of the MSE before and after the correction of the national summer precipitation forecast (puzzle).

**FIGURE 9:**
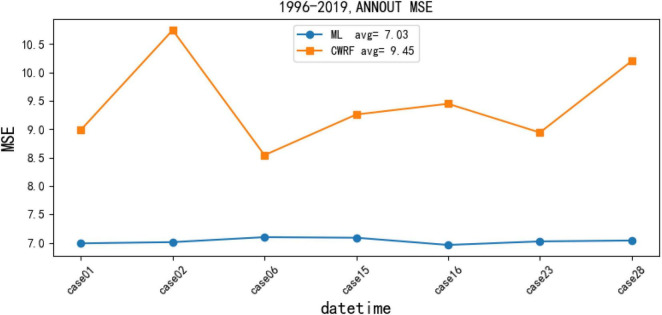
Mean square error (MSE) change graph of precipitation data correction results for 7 cases (i.e., 01, 02, 06, 15, 16, 23, and 28) of CWRF based on the artificial DD model.

Figure 9 contains the comparison of two calculation results, namely, (1) MSE change (CWRF) between simulated precipitation (case) and actual observed precipitation (OBS) in CWRF model and (2) use artificial network to correct precipitation and actual observation (OBS) between MSE change (ML) under the corresponding parameters; from Figure 9, it can be seen that there are fluctuations between the different CWRF model data and the MSE of the actual observation data, indicating that different physical parameter configurations have different degrees of error in the simulated flood season precipitation; after correction, the rainy season precipitation of the next 7 cases all increased to varying degrees. The 7 case results of CWRF simulated precipitation, respectively, calculate the MSE, and the average MSE is 9.45, and the corrected average MSE is 7.03, a decrease of 2.42 (equivalent to a 26% decrease in MSE). It can be seen from the figure that the MSE of the CWRF model rainy season precipitation data fluctuates greatly, and the corrected 7 case results have stabilized, indicating that the artificial network model has improved the accuracy of the model forecast data to a certain extent. The forecasting performance of CWRF has to be improved.

(2) Comparison of the TCC before and after the correction of the national summer precipitation forecast (puzzle).

**FIGURE 10:**
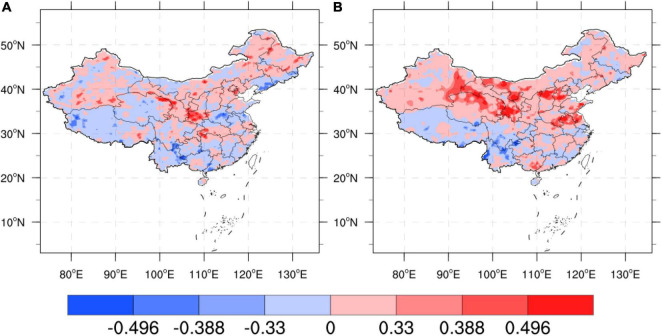
Comparison of TCC before and after CWRF prediction correction for 7 case sets, **(A)** CWRF prediction and **(B)** machine learning correction, the color code number is the same as Figure 4.

Figure 10 shows the TCC comparison before and after the correction of CWRF forecasts for 7 cases and 4 time-time sets (28 samples in total). It can be seen from the figure that there are fewer positive correlation areas for TCC before the correction, especially in eastern China. There is a large area of negative value in the key area (the Yangtze River Basin to SCH). After the correction of the artificial network model, the TCC of the key precipitation area in eastern China shows a large positive correlation, especially in the south of the Yangtze River and the northeast. After correction by machine learning, the number of positive TCC regions and significant regions increased significantly across the country, especially in the Yangtze River Basin and Northeast China, indicating that the ANN-DD algorithm model can achieve certain corrections to the national regional precipitation forecast data during the flood season. Forecasting skills have been significantly improved.

(3) Comparison of the spatial correlation coefficient (ACC) before and after the correction of the national summer precipitation forecast (puzzle).

**FIGURE 11:**
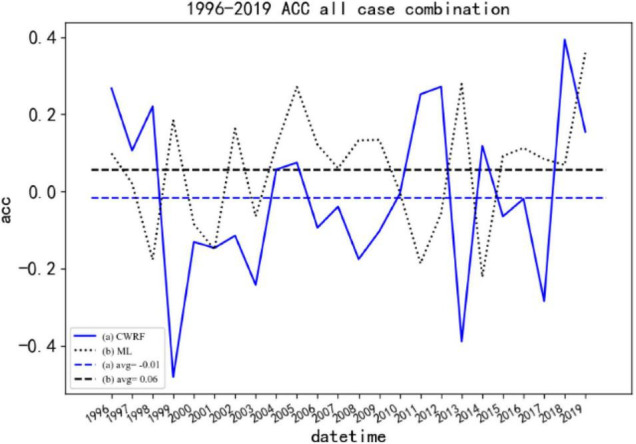
Comparison of ACC before and after CWRF forecast correction for 7 case sets. (a) CWRF forecast, (b) machine learning correction, and (a, b) 1996–2019 average.

Figure 11 shows the ACC comparison before and after the correction of CWRF forecasts for 7 cases and 4 time sets (28 samples in total). It can be seen from the figure that the average ACC before correction is −0.01, and the ACC value in most years is low at 0, there is a negative correlation. After correction by machine learning, the ACC value has increased significantly. The correction result of the ML (curve b in the figure) scheme is about 0.1 higher than before the correction, and the ACC value in most years is greater than 0. At the same time, the predicted result after correction can be seen in the figure. The stability has improved, and the fluctuations before correction are large, showing a good correction effect. It shows that the ANN algorithm model can make certain corrections to the national regional rainy season precipitation forecast data, and the forecasting skills have improved.

(4) Comparison of comprehensive trend anomaly scores (Ps) before and after correction of the national summer precipitation forecast (puzzle).

**FIGURE 12:**
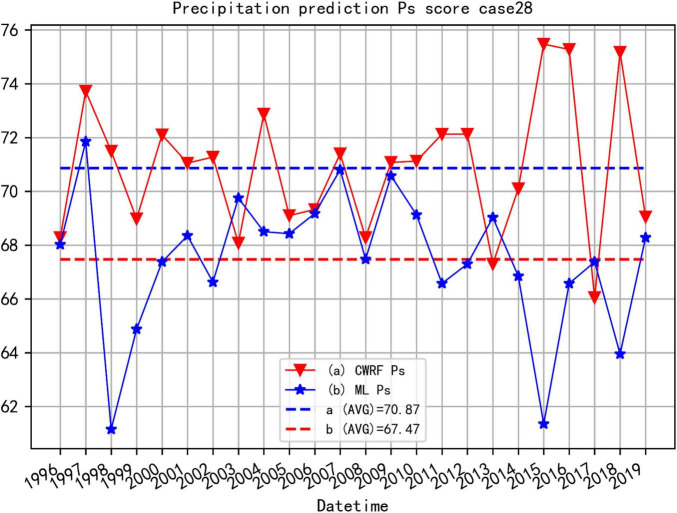
Comparison of Ps before and after CWRF forecast correction for the 4 time-time sets of case 28. (a) CWRF forecast, (b) machine learning correction, and (a, b) mean Ps from 1996 to 2019.

**FIGURE 13:**
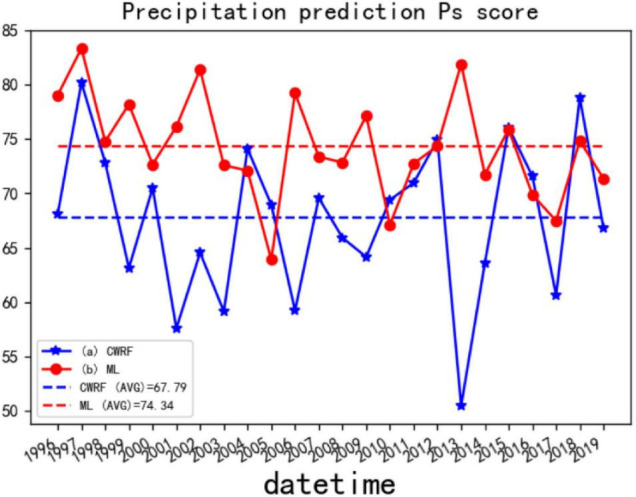
Comparison of Ps before and after CWRF forecast correction for 7 case sets. (a) CWRF forecast, (b) machine learning correction, and (a, b) mean Ps from 1996 to 2019.

Consider case 28 (Figure 12) and 7 case set (Figure 13) as examples to illustrate the improvement effect of the CWRF model prediction Ps of the machine learning correction method.

Figure 12 shows the comparison of the CWRF prediction and the Ps score corrected by ANN after the 4 time collections of case 28 (the 4 time precipitation predictions of case 01 are collected before calculating the Ps). It can be seen from the figure that the average value of Ps before correction is around 70.87, and after correction by machine learning, the Ps score is around 72.55, and the overall prediction skills have improved. Among them, ML (curve b in the figure) has a better overall trend abnormality score in 1996–2014 than CWRF’s original prediction (curve a in the figure); the Ps score in 2015–2019 fluctuates more. This means that the simulated precipitation of case 28 is better because of machine learning.

Figure 13 shows the comparison of CWRF prediction and ANN corrected Ps after the collection of 4 starting times for 7 cases (before calculating Ps, the precipitation predictions for 4 times (28 samples in total) of 7 cases are collected) deal with. The CWRF forecast on the graph shows that the average Ps score from 1996 to 2019 is about 67.79; the average Ps score after machine learning correction is about 74.34; Figure 13 generally shows the same correction effect as Figure 12, except for the Ps value of individual years’ difference. This shows that the precipitation forecasting skills during the flood season have improved to a certain extent compared with before the correction, and the Ps score of most years after the correction has improved. The Ps score shows a stable trend with a small fluctuation range. Based on the above two calculations of Ps anomaly scores for different physical parameter configurations and integrations, it can be concluded that the prediction performance of the revised algorithm model based on machine learning for the rainy season precipitation prediction results is improved compared with the climate model simulation, which can achieve a certain degree of improvement in the rainy season precipitation forecast skills.

In summary, it can be concluded from Figure 13 that the MSE, Ps, ACC, and TCC values before and after the CWRF prediction are improved, and the prediction performance of the summer precipitation prediction result correction algorithm model based on the dendritic neural network model can be obtained. Compared with the CWRF climate model simulation, it is improved and can realize the correction of summer precipitation forecast data to a certain extent.

## Conclusion and Future Work

In this article, we used the information from nearby regions and time series and latitude and longitude positions to organize the data and then divide it into eight regions according to the climate characteristics of different regions. Then, the ANN model is improved, and the dendritic module is introduced to improve the generalization ability of the model. When constructing the network model in this article, the number of hidden layers is 2, and the number of neurons in each layer is 9. The back propagation algorithm is used for model training. Through cross-validation experiments on the model, it is found that the activation function of the hidden layer is adopted by the RELU function. The optimization algorithm adopts the Adam algorithm model to predict better. The CWRF regional climate model simulated summer precipitation was corrected by optimizing the relevant meteorological elements such as precipitation and temperature and analyzed with related evaluation indicators such as MSE, TCC, and ACC. It was found that all three indicators were improved, indicating that the artificial dendritic network model is effective for CWRF. The accuracy of model forecast data has been improved, which can improve the forecasting performance of CWRF to a certain extent. The experimental results are obtained using the cross-validation method, which can objectively evaluate the generalization performance of the model. Experiments show that good results can be achieved, which makes this method a good choice for the meteorological field.

This article mainly discusses the problem of precipitation correction in flood season. In theory, this idea and method can be used in other related meteorological forecasting fields and can be used as future work. At present, the model only considers the output of the climate model, and many other related elements are not used for modeling, so the advantages of big data are not fully utilized. In the future, more meteorological elements can be introduced to overcome this limitation. This article cannot remove the impact of extreme weather precipitation data when using precipitation data, and only the forecast output of the meteorological model is used as the modeling object of the algorithm in this article. Taking into account the complexity of meteorological problems. Furthermore, we hope to reduce the impact of extreme weather by layered modeling of precipitation. Other data, such as relevant observation data, can be added as model inputs to check whether the accuracy of the model can be improved.

## Data Availability Statement

The data analyzed in this study is subject to the following licenses/restrictions: It is the business data provided by the relevant departments of the China Meteorological Administration, non-public data, and needs to be applied to the relevant departments of the China Meteorological Administration. Requests to access these datasets should be directed to https://data.cma.cn/.

## Author Contributions

TL and CQ responsible for designing and implementing algorithms. LW and JC responsible for organize experiment. YR responsible for the analysis of experimental results and algorithm optimization. JC responsible for language polish. All authors contributed to the article and approved the submitted version.

## Conflict of Interest

The authors declare that the research was conducted in the absence of any commercial or financial relationships that could be construed as a potential conflict of interest. The handling editor declared a shared affiliation with the authors [CQ, TL, LW, JC, and YR] at the time of review.

## Publisher’s Note

All claims expressed in this article are solely those of the authors and do not necessarily represent those of their affiliated organizations, or those of the publisher, the editors and the reviewers. Any product that may be evaluated in this article, or claim that may be made by its manufacturer, is not guaranteed or endorsed by the publisher.
